# The relationship between anxiety and internet gaming disorder in children during COVID-19 lockdown: a network analysis

**DOI:** 10.3389/fpsyt.2023.1144413

**Published:** 2023-05-17

**Authors:** Tianqi Yang, Yang He, Chunyan He, Yueqi Yang, Lin Wu, Bin Wei, Ruina Dong, Mengyuan Yang, Zhaojun Pu, Saiming Wang, Jing Li, Tao Xu, Xufeng Liu, Shengjun Wu

**Affiliations:** ^1^Department of Military Medical Psychology, Air Force Medical University, Xi’an, China; ^2^Department of Nursing, Air Force Medical University, Xi’an, China; ^3^Xiong’an Rongxi Linquan Primary School, Xiong’an New Area, China; ^4^Xijing Hospital, Air Force Medical University, Xi’an, China; ^5^Tangdu Hospital, Air Force Medical University, Xi’an, China; ^6^The First Primary School of Fuliang County, Jingdezhen, China; ^7^School of Public Health, Xi’an Medical University, Xi’an, China; ^8^Psychology Section, Secondary Sanatorium of Air Force Healthcare Center for Special Services, Hangzhou, China

**Keywords:** anxiety, internet gaming disorder, children, COVID-19, network analysis

## Abstract

**Background:**

Internet gaming disorder (IGD) has become a social problem in children. Evidence from previous studies has proven that anxiety is associated with IGD. However, IGD was always assessed as a whole based on total scores, and the fine-grained relationship between anxiety and IGD was hidden.

**Objective:**

The present study aims to investigate the fine-grained relationship between anxiety and IGD in elementary school students during the COVID-19 lockdown, and to identify potential targets for psychological interventions.

**Methods:**

During the lockdown caused by the COVID-19 pandemic, 667 children from a primary school in China were investigated by the Spence Children’s Anxiety Scale-Short Version and Internet Gaming Disorder Scale. R4.1.1 software was used to construct a network model, assess bridge centrality, and test the robustness of the network and conduct a network.

**Results:**

There were 23 cross-community edges (weight ranged from −0.03 to 0.12), and each node of anxiety was connected to different nodes of IGD. The nodes with the top 80th percentile bridge expected influence were A2 “social phobia” (0.20), A3 “panic disorder” (0.21) and IGD5 “escape” (0.22). The robustness of the network was acceptable.

**Conclusion:**

From the perspective of network analysis, the present study explored the correlation pathways between anxiety and IGD in children and identified social phobia and panic disorder as the best targets for intervention to reduce IGD.

## 1. Introduction

The 2020 national research report on internet access by minors shows that with the rapid development of new internet technologies, internet access by minors is becoming increasingly common. By the end of 2019, 175 million Chinese minors were internet users, accounting for 93.1% of the total number of minors, while the internet penetration rate of elementary school students was also high, at 89.4% (1). There is a trend of younger teenagers accessing the internet (2). Meanwhile, the childhood stage is a critical period for psychological development and personality formation (3). Children are curious about new things, good at imitation (4, 5), undisciplined and easily impulsive (6, 7). Therefore, children are more likely to be addicted to the internet than more mature individuals (8), and one of the main types of internet addiction is internet gaming disorder (IGD) (9). IGD is described as being addicted to online games for a long time and developing a strong sense of dependence and continuous craving behavior (10, 11).

The current diagnosis and definition of IGD mainly relies on the fifth edition of the Diagnostic and Statistical Manual of Mental Disorders (DSM-5) published by the American Psychiatric Association in 2013 (12), which lists nine diagnostic criteria for IGD: addiction to games; increased tolerance; giving up other activities to play games; reckless disregard for the adverse effects; using games as a way to cope and escape negative emotional experiences; social/occupational disruptions because of playing games; trying and failing to control online game time; deliberately hiding online game-related problems; and becoming irritable and temperamental if they cannot access the internet. During the outbreak of COVID-19, school closures, prolonged isolation at home, and reduced offline interactions increases the risk of IGD (13). IGD causes psychological and physical damage to children (14–17), jeopardizes social skills and academic performance (18), and even leads to suicide and delinquency (19–21). In light of these findings, there is an urgent need to explore the mechanisms of IGD in elementary school students to provide targets for early psychological intervention.

Evidence from previous studies has proven that anxiety is associated with IGD (22, 23). Individuals with anxiety disorders are more likely to socialize online, overuse smartphones and be addicted to online games (24–26). Currently, influenced by all kinds of media information and uncertainty about the perceived risk of the epidemic, people are more anxious in the face of the COVID-19 lockdown. Therefore, the relationship between anxiety and IGD during the pandemic has attracted the attention of researchers. Fazeli et al. (13) found that IGD significantly influenced anxiety and depression symptoms in adolescents during the COVID-19 epidemic. Wang et al. (27) found that adolescents with anxiety disorders were more likely to develop IGD in the presence of fear of missing out. Elhai et al. (28) found that COVID-19 anxiety was strongly associated with smartphone use.

However, in previous studies investigating the relationship between anxiety and IGD, IGD was always assessed as a whole based on total scores (29). Indeed, the DSM-5 diagnostic criteria for IGD contain nine heterogeneous symptoms, and each symptom may be sensitive to specific risk factors and represent a unique risk pathway mechanism. The correlation based on the total score hides the fine-grained relationship between the different symptoms of anxiety and IGD (30). In addition, previous correlation studies cannot provide intervention targets at symptom level because there was no measure that quantifies the relative importance of items.

Network analysis is a promising statistical method to solve these problems. As data-driven processing that does not rely on a priori assumptions about the relationships between variables (31), network analysis helps reveal the relationships between psychological variables at the fine-grained level. The network structure consists of nodes representing psychological variables and lines representing statistical relationships between variables (32). Compared with traditional statistical correlation research, network analysis has the following advantages: (a) the ability to clarify fine-grained relationships between variables (33); (b) the potential to avoid spurious correlations due to a large number of variables (34); (c) visualization of association patterns between different variables (35); and (d) the capacity to assess the relative importance of variables by providing a centrality index (36). The term community is used to denote a theory-based set of psychological variables, and index bridge centrality helps to accurately capture the variables that play a key role in bridging communities (37), these bridge variables are often considered as potential targets for psychological intervention. Thus, network analysis may help deepen our understanding of the relationship between anxiety and IGD and provide targets for psychological intervention.

In summary, based on network analysis, the present study investigated the fine-grained relationship between anxiety and IGD in elementary school students during the COVID-19 lockdown. We developed a network model to explore the correlation pathways between the symptoms of anxiety and IGD and assessed bridge centrality to provide a theoretical basis for identifying effective symptom targets for psychological interventions.

## 2. Materials and methods

### 2.1. Participants

In April 2022, during the lockdown caused by the COVID-19 pandemic, a cluster sample of children in Baoding City, China, was investigated. Inclusion criteria: (a) children from the elementary grades of primary school; (b) isolated at home for at least 1 week due to the COVID-19 epidemic; (c) clear consciousness and normal perceptual function; (d) volunteered to participate in the study and provided the informed consent. Exclusion criteria: (a) cognitive dysfunction or communication disorder; (b) incomplete questionnaires. The Spence Children’s Anxiety Scale-Short Version (SCAS-S) and Internet Gaming Disorder Scale (IGDS) powered by www.wjx.cn were used to survey the children after explaining the purpose and method of the research to the children and their parents. The parents were required to read the questions and response options of each item to their children, and then the children answered according to their own feelings. A total of 722 questionnaires were distributed, and incomplete questionnaires were deemed invalid. Finally, 667 valid questionnaires were collected for an effective recovery rate of 92.38%. The present research followed the Helsinki Declaration and was approved by the Ethics Committee of Xijing Hospital of Air Force Medical University (Project No. CHiCTR1800019761).

### 2.2. Measures

#### 2.2.1. SCAS-S

The SCAS-S was revised by Ahlen et al. (38) on the basis of the Spence Children’s Anxiety Scale (39) to evaluate anxiety symptoms. The SCAS-S contains 19 items and consists of 5 dimensions, namely, separation anxiety, social phobia, panic disorder, physical injury fear and generalized anxiety. The SCAS-S is a Likert 4-point scoring scale: 0 = never, 1 = rarely, 2 = sometimes, 4 = always. The higher the score, the more severe the anxiety. The Cronbach’s α of this scale in the present research was 0.80.

#### 2.2.2. IGDS

The IGDS was compiled by Pontes and Griffiths (40) to assess IGD in the previous 6 months. The Chinese version of the IGDS revised by Jiang and Zeng (41) was used in this study. The nine items of the IGDS correspond to the nine diagnostic criteria for online game addiction in DSM-5, namely, preoccupation, tolerance, giving up other activities, continuing despite problems, escape, negative consequences, loss of control, deception, and withdrawal. IGDS is measured on a Likert 5-point scoring scale: 1 = never, 2 = often not, 3 = sometimes, 4 = often, and 5 = always. The higher the score, the stronger the tendency of IGD. The Cronbach’s α of this scale in the present research was 0.89.

### 2.3. Statistical analysis

Statistics of demographic characteristics and scale scores were calculated by SPSS 22.0 software. Network model construction, bridge centrality analysis, and robustness tests were conducted by R4.1.1 software.

#### 2.3.1. Network model construction

The Gaussian graphical models (GGMs) were fitted to the data (32). In the network model, nodes represented variables of SCAS-S and IGDS and were divided into anxiety community (separation anxiety, social phobia, panic disorder, physical injury fear, and generalized anxiety) and IGD community (preoccupation, tolerance, giving up other activities, continuing despite problems, escape, negative consequences, loss of control, deception, and withdrawal). Edges between nodes represented partial correlations after statistically eliminating interference from all other nodes (34). Edge color indicates the nature of the partial correlation, red edges suggest a negative correlation, blue edges suggest a positive correlation, edge saturation indicates the intensity of the partial correlation, and a more saturated edge suggests a larger partial correlation. The combination of least absolute shrinkage and selection operator (LASSO) (42) regularization and extended Bayesian information criterion (EBIC) (43) was used in network model construction to shrink small edges to zero weight and make the network stable and clear (44). To balance the sensitivity and specificity (45), the EBIC hyperparameter γ was set to 0.5. The Fruchterman-Reingold algorithm was used to build the network layout (46). The anxiety-IGD network model was constructed with the qgraph package (47).

#### 2.3.2. Bridge centrality analysis

In the present study, the bridge expected influence (BEI) was estimated. As a kind of bridge centrality index, the BEI of a node is defined as the sum of the edge weights between the node and all nodes from other communities (37). In a bidirectional network, a higher value of BEI indicates a greater likelihood of affecting or being affected by other communities. The bridge centrality of nodes was evaluated by the networktools package (37).

#### 2.3.3. Network robustness test

To test the network robustness, three operations were conducted. First, we estimated the 95% confidence interval of edge weights to evaluate the accuracy by non-parametric bootstrapping (1,000 bootstrapped samples). A relatively narrow 95% confidence interval ensures adequate accuracy of the edge weights (44). Second, we tested the stability of BEI by case-dropping bootstrapping (1,000 bootstrapped samples). We also calculated the correlation stability coefficient (CS-coefficient) to quantify the stability; a value larger than 0.5 of CS-coefficient indicates ideal stability (44). Finally, we tested the difference in the BEI indices of nodes and the difference in the edge weights of node pairs by bootstrapping (1,000 bootstrapped samples, α = 0.05). The network robustness was tested with the bootnet package (44).

## 3. Results

### 3.1. Demographic characteristics and descriptive statistics

The demographic characteristics of the sample are displayed in Table 1. The means and standard deviations of the variables in the anxiety-IGD network are displayed in Table 2.

**TABLE 1:** Demographic characteristics of the sample (*n* = 667).

Variables	Mean (SD), Range, %
Age	7.63 (0.65), 6−10
Male gender	396 (59.37%)
Educational level	
First grade	260 (38.98%)
Second grade	407 (61.02%)
Total anxiety score	13.73 (8.55), 0−46
Total score of IGD	13.59 (6.10), 9−42

**TABLE 2:** The means and standard deviations of variables in the anxiety-IGD network.

Variables	Mean (SD)
**Dimensions of anxiety**	
A1 separation anxiety	3.69 (2.43)
A2 social phobia	3.32 (2.35)
A3 panic disorder	1.05 (1.84)
A4 physical injury fear	2.70 (2.29)
A5 generalized anxiety	2.97 (2.50)
**Items of IGD**	
IGD1 preoccupation	1.69 (1.05)
IGD2 tolerance	1.78 (1.08)
IGD3 giving up other activities	1.38 (0.83)
IGD4 continuing despite problems	1.50 (0.89)
IGD5 escape	1.33 (0.81)
IGD6 negative consequences	1.38 (0.82)
IGD7 loss of control	1.52 (1.00)
IGD8 deception	1.50 (0.95)
IGD9 withdrawal	1.50 (0.99)

### 3.2. Fine-grained relationship between anxiety and IGD

**FIGURE 1:**
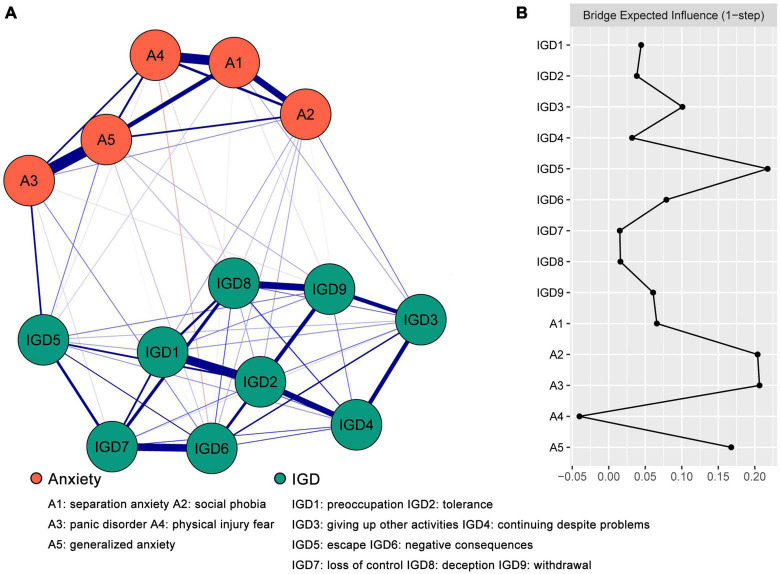
The anxiety-IGD network model and bridge expected influence (BEI). **(A)** The anxiety-IGD network model. The red edges suggested a negative correlation, while the blue edges suggested a positive correlation, and a more saturated edge suggested a larger partial correlation. **(B)** The bridge expected influence indices of the nodes in the network (raw score).

The anxiety-IGD network is displayed in Figure 1A. Logically, there was a maximum of 45 edges across the communities in the network; in the present research, there were 23 cross-community edges (weights ranged from −0.03 to 0.12). Overall, positive edges accounted for the vast majority of cross-communities edges (86.96%). The positive cross-community edges contained A1 “separation anxiety”—IGD3 “giving up other activities” (edge weight = 0.04), A1 “separation anxiety”—IGD5 “escape” (edge weight = 0.02), A1 “separation anxiety”—IGD9 “withdrawal” (edge weight = 0.01), A2 “social phobia”—IGD1 “preoccupation” (edge weight = 0.04), A2 “social phobia”—IGD2 “tolerance” (edge weight = 0.04), A2 “social phobia”—IGD3 “giving up other activities” (edge weight = 0.06), A2 “social phobia”—IGD4 “continuing despite problems” (edge weight = 0.01), A2 “social phobia”—IGD6 “negative consequences” (edge weight = 0.04), A2 “social phobia”—IGD8 “deception” (edge weight = 0.02), A3 “panic disorder”—IGD5 “escape” (edge weight = 0.12), A3 “panic disorder”—IGD6 “negative consequences” (edge weight = 0.06), A3 “panic disorder”—IGD7 “loss of control” (edge weight = 0.02), A3 “panic disorder”—IGD9 “withdrawal” (edge weight = 0.01), A4 “physical injury fear” —IGD5 “escape” (edge weight = 0.01), A5 “generalized anxiety”—IGD1 “preoccupation” (edge weight = 0.003), A5 “generalized anxiety”—IGD4 “continuing despite problems” (edge weight = 0.03), A5 “generalized anxiety”—IGD5 “escape” (edge weight = 0.06), A5 “generalized anxiety”—IGD6 “negative consequences” (edge weight = 0.03), A5 “generalized anxiety”—IGD8 “deception” (edge weight = 0.01) and A5 “generalized anxiety”—IGD9 “withdrawal” (edge weight = 0.04). The negative cross-community edges contain A1 “separation anxiety”—IGD6 “negative consequences” (edge weight = −0.01), A4 “physical injury fear”—IGD6 “negative consequences” (edge weight = −0.03) and A4 “physical injury fear”—IGD8 “deception” (edge weight = −0.02). The correlation matrix of the network model can be found in Supplementary Table 1.

The accuracy test of edge weights is displayed in Supplementary Figure 1. The 95% confidence interval of edge weights in the anxiety-IGD network was relatively narrow, indicating that the edge estimation was accurate. The difference test of edge weights is displayed in Supplementary Figure 2.

### 3.3. Bridges between anxiety and IGD

The BEI is displayed in Figure 1B. The nodes with the top 80th percentile BEI were A2 “social phobia” (0.20), A3 “panic disorder” (0.21) and IGD5 “escape” (0.22). As shown in Supplementary Figure 3, the BEI indices of A2 “social phobia,” A3 “panic disorder” and IGD5 “escape” were statistically larger than those of most other nodes. As described in Supplementary Figure 4, the average correlation of BEI indices of the subsample and the original sample showed a relatively gentle downward trend with the reduction of subsample. The value of the CS-coefficient on the BEI was 0.52, which was larger than 0.5, indicating adequate stability.

## 4. Discussion

Based on network analysis, the present study revealed the correlation pathways between anxiety and IGD in elementary school children during the COVID-19 lockdown and provided suggestions for IGD interventions through the evaluation of the BEI index. The stability and accuracy of the network model were acceptable, which guaranteed the reliability of our conclusion.

### 4.1. Correlation pathways between anxiety and IGD

The detailed relationships between variables revealed by network analysis can provide indications of the correlation pathways through which different aspects of anxiety affect IGD (48, 49). These correlation pathways may represent underlying interaction mechanisms of anxiety and IGD. In light of this, we discussed the most powerful pathways bridging various aspects of anxiety and contents in IGD.

Among the four pathways through which “separation anxiety” correlated with IGD, the strongest pathway was “separation anxiety”—“giving up other activities,” while among the six pathways through which “social phobia” correlated with IGD, the strongest pathway was “social phobia”—“giving up other activities.” Separation anxiety is described as the irrational fear or anxiety of being separated from family or close attachment (12, 50). According to Bowlby’s attachment theory, the quality of parental care a child receives in early years has profound effects on future mental health (51). Children who do not receive effective care and attention from parents are incapable of dealing with separation anxiety. Many children have been separated from their relatives and even parents due to the COVID-19 lockdown, and separation anxiety caused by the disruption of close relationships drives children to online games, which may meet their emotional requirements. Social phobia is described as the excessive fear of social occasions and interactions with others and may result in social isolation (52). Baker and Hudson (53) found that children with social phobia had narrower social networks and poorer friendship quality and were more likely to experience social alienation than children with other anxiety symptoms. This may be due to increased sensitivity to social exclusion and social withdrawal in adolescents with symptoms of social phobia leading to deficits in interpersonal skills (54). Therefore, children with social phobia tend to abandon offline social activities and indulge in virtual worlds. The reason is that the internet provides a platform for them to communicate anonymously.

Among the four pathways through which “panic disorder” correlated with IGD, the strongest pathway was “panic disorder”—“escape,” while among the six pathways through which “generalized anxiety” correlated with IGD, the strongest pathway was “generalized anxiety”—“escape.” Panic disorder is characterized by misinterpretations of explosive sensory and emotion, i.e., heart palpitations, dyspnea, dizziness, and near-death experience (55, 56). Inconsistent with panic disorder, generalized anxiety is characterized by persistent, relatively mild symptoms on most days for at least 6 months, such as nervosity, irritability, fatigue, attention and memory problems, and insomnia (12, 57, 58). Both panic disorder and generalized anxiety lead to evident negative emotional experiences, and when there is no appropriate outlet, such as during the COVID-19 lockdown, children often turn to online games to escape these emotional experiences.

Among the three pathways through which “physical injury fear” correlated with IGD, the strongest pathway was “physical injury fear”—“negative consequences.” The symptoms of physical injury fear are similar to those of specific phobia; they all manifest as extreme and persistent fear of a certain object or situation, such as heights, tunnels, darkness, or worms (59). COVID-19 may cause death and sequelae (60), and related reports have led to increased sensitivity to physical injury fear. In addition, the children in the present study were too young to identify the authenticity of information, so they were excessively afraid of dangerous information and were prone to online gaming addiction, which could lead to a series of negative consequences.

### 4.2. Optimal targets for intervention

Anxiety is comorbid with IGD (61), and the activation of bridge symptoms can increase the risk of transferring from one disorder to another (37). In network analysis, bridge centrality explained the comorbidity and reciprocity of different disorders (62). The current study identified bridge symptoms between anxiety and IGD in children. Bridge symptom identification helps reveal fecund information on comorbidities and provide prioritized clinical targets to prevent co-occurrence (63, 64). The BEI indices of “social phobia” and “panic disorder” were the largest in the anxiety community. From the perspective of the network, the intervention of “social phobia” and “panic disorder” has a greater impact on IGD than other nodes of the anxiety community, so they are potential best targets for interventions. Previous studies have confirmed the relationship between social phobia and IGD (65). In fact, children with social phobia have more personal space and time, which is a crucial risk factor for IGD (66). Given the acuteness and recurrence of panic disorder symptoms (67), individuals may have persistent worries, and online games may provide a distraction. Gaming online is an escape strategy to alleviate anxious emotions, and people may spend excessive amounts of time on online games as a coping mechanism to escape from the reality of their worries and difficulties (24–26). However, online gaming does not actually solve the problem, and wasting too much time on games makes things worse. A vicious cycle may further exacerbate psychological distress in people with IGD. Therefore, there is a need to implement targeted interventions to improve intervention efficiency and save medical resources.

Many intervention methods have been proven effective for anxiety. Cognitive behavioral therapy is the most experienced and effective psychosocial therapy for treating social phobia in adults and children (68); furthermore, the well-known Gestalt therapy is also effective in reducing anxiety in elementary school children (69). Finally, parent–child interaction therapy can prevent IGD by reducing the child’s level of anxiety by establishing a good parent–child relationship (70, 71).

### 4.3. Limitations

Several limitations should be pointed out in the present study. First, since cross-sectional data were used in our research and the relationships in networks were bidirectional, the determination of causality needs further support from longitudinal research. Second, the sample scope was limited to students in elementary school. Although we aimed to detect and intervene as early as possible, the results may not be suitable for generalization to older children. Finally, there was no control group, and the comparison of networks of students in lockdown and those in school may provide more meaningful findings.

## 5. Conclusion

In conclusion, the visual network structure provided a delicate description of the correlation pathways between anxiety and IGD; the BEI comparison helped to determine the bridges between anxiety and IGD and recommended social phobia and panic disorder as the potential targets for intervention of IGD. This study represents the first application of network analysis to explore the relationships between anxiety and IGD in children during COVID-19 lockdown and provides a reliable reference for the practice of psychological intervention.

## Data availability statement

The raw data supporting the conclusions of this article will be made available by the authors, without undue reservation.

## Ethics statement

The studies involving human participants were reviewed and approved by the Ethics Committee of Xijing Hospital of Air Force Medical University (Project No. CHiCTR1800019761). Written informed consent to participate in this study was provided by the participants’ legal guardian/next of kin.

## Author contributions

TY, SJW, TX, and BW: concept and design. YY, SMW, RD, and ZP: acquisition of the data. TY, JL, and MY: analysis and interpretation of the data. TY, YH, CH, and LW: drafting of the manuscript. XL and SJW: critical revision of the manuscript. All authors contributed to the article and approved the submitted version.

## Supplementary material

Supplementary Figure 1 Accuracy test of edge weights in the anxiety-IGD network. The red line indicates the sample edge weight values and the gray area indicates the bootstrapped confidence intervals. A1, separation anxiety; A2, social phobia; A3, panic disorder; A4, physical injury fear; A5, generalized anxiety; IGD1, preoccupation; IGD2, tolerance; IGD3, giving up other activities; IGD4, continuing despite problems; IGD5, escape; IGD6, negative consequences; IGD7, loss of control; IGD8, deception; IGD9, withdrawal.

Supplementary Figure 2 Bootstrapped difference test of edge weights in the anxiety-IGD network. The black box indicates that the edge weights of the two corresponding node pairs have a significant difference, the gray box indicates no significant difference. Blue and red boxes on the diagonal correspond to edge weights with positive and negative correlations, respectively. A1, separation anxiety; A2, social phobia; A3, panic disorder; A4, physical injury fear; A5, generalized anxiety; IGD1, preoccupation; IGD2, tolerance; IGD3, giving up other activities; IGD4, continuing despite problems; IGD5, escape; IGD6, negative consequences; IGD7, loss of control; IGD8, deception; IGD9, withdrawal.

Supplementary Figure 3 Bootstrapped difference test of bridge expected influences in the anxiety-IGD network. The black box indicates that the bridge expected influences of the two corresponding nodes have a significant difference, the gray box indicated no significant difference. A1, separation anxiety; A2, social phobia; A3, panic disorder; A4, physical injury fear, A5, generalized anxiety, IGD1, preoccupation; IGD2, tolerance; IGD3, giving up other activities; IGD4, continuing despite problems; IGD5, escape; IGD6, negative consequences; IGD7, loss of control; IGD8, deception; IGD9, withdrawal.

Supplementary Figure 4 Stability of bridge expected influences in the anxiety-IGD network. The red bar represents the average correlation between bridge expected influences in the full sample and subsample with the red area depicting the 2.5th quantile to the 97.5th quantile.
