# Supplementary material for: The relationship between anxiety and internet gaming disorder in children during COVID-19 lockdown: a network analysis
Source: Front Psychiatry. 2023 May 17;14:1144413. doi: 10.3389/fpsyt.2023.1144413 (PMC10229880; doi:10.3389/fpsyt.2023.1144413)
Supplement: Supplementary file 1 [file Table_1.docx]

Table S1 [Correlation](javascript:;) [Matrix](javascript:;) of the anxiety-IGD network model

|  | IGD1 | IGD2 | IGD3 | IGD4 | IGD5 | IGD6 | IGD7 | IGD8 | IGD9 | A1 | A2 | A3 | A4 | A5 |
| --- | --- | --- | --- | --- | --- | --- | --- | --- | --- | --- | --- | --- | --- | --- |
| IGD1 |  | 0.3070 | 0.0512 | 0.0702 | 0.0436 | 0.0028 | 0.1243 | 0.1371 | 0.0580 | 0.0000 | 0.0412 | 0.0000 | 0.0000 | 0.0032 |
| IGD2 | 0.3070 |  | 0.0642 | 0.2310 | 0.1258 | 0.0486 | 0.0659 | 0.0406 | 0.1786 | 0.0000 | 0.0384 | 0.0000 | 0.0000 | 0.0000 |
| IGD3 | 0.0512 | 0.0642 |  | 0.1874 | 0.0337 | 0.1152 | 0.0163 | 0.0672 | 0.1842 | 0.0392 | 0.0617 | 0.0000 | 0.0000 | 0.0000 |
| IGD4 | 0.0702 | 0.2310 | 0.1874 |  | 0.0479 | 0.0826 | 0.0803 | 0.0994 | 0.0620 | 0.0000 | 0.0053 | 0.0000 | 0.0000 | 0.0266 |
| IGD5 | 0.0436 | 0.1258 | 0.0337 | 0.0479 |  | 0.1019 | 0.1466 | 0.0000 | 0.0629 | 0.0204 | 0.0000 | 0.1228 | 0.0109 | 0.0635 |
| IGD6 | 0.0028 | 0.0486 | 0.1152 | 0.0826 | 0.1019 |  | 0.2737 | 0.0775 | 0.1493 | -0.0067 | 0.0355 | 0.0569 | -0.0329 | 0.0260 |
| IGD7 | 0.1243 | 0.0659 | 0.0163 | 0.0803 | 0.1466 | 0.2737 |  | 0.1595 | 0.0000 | 0.0000 | 0.0000 | 0.0151 | 0.0000 | 0.0000 |
| IGD8 | 0.1371 | 0.0406 | 0.0672 | 0.0994 | 0.0000 | 0.0775 | 0.1595 |  | 0.2571 | 0.0000 | 0.0219 | 0.0000 | -0.0181 | 0.0123 |
| IGD9 | 0.0580 | 0.1786 | 0.1842 | 0.0620 | 0.0629 | 0.1493 | 0.0000 | 0.2571 |  | 0.0129 | 0.0000 | 0.0117 | 0.0000 | 0.0361 |
| A1 | 0.0000 | 0.0000 | 0.0392 | 0.0000 | 0.0204 | -0.0067 | 0.0000 | 0.0000 | 0.0129 |  | 0.2478 | 0.0151 | 0.3314 | 0.1932 |
| A2 | 0.0412 | 0.0384 | 0.0617 | 0.0053 | 0.0000 | 0.0355 | 0.0000 | 0.0219 | 0.0000 | 0.2478 |  | 0.0504 | 0.1432 | 0.1257 |
| A3 | 0.0000 | 0.0000 | 0.0000 | 0.0000 | 0.1228 | 0.0569 | 0.0151 | 0.0000 | 0.0117 | 0.0151 | 0.0504 |  | 0.1223 | 0.3751 |
| A4 | 0.0000 | 0.0000 | 0.0000 | 0.0000 | 0.0109 | -0.0329 | 0.0000 | -0.0181 | 0.0000 | 0.3314 | 0.1432 | 0.1223 |  | 0.1300 |
| A5 | 0.0032 | 0.0000 | 0.0000 | 0.0266 | 0.0635 | 0.0260 | 0.0000 | 0.0123 | 0.0361 | 0.1932 | 0.1257 | 0.3751 | 0.1300 |  |

Note: A1 = separation anxiety, A2 = social phobia, A3 = panic disorder, A4 = physical injury fear, A5 = generalized anxiety, IGD1 = preoccupation, IGD2 = tolerance, IGD3 = giving up other activities, IGD4 = continuing despite problems, IGD5 = escape, IGD6 = negative consequences, IGD7 = loss of control, IGD8 = deception, IGD9 = withdrawal.
