# Supplementary figures and images for: The relationship between anxiety and internet gaming disorder in children during COVID-19 lockdown: a network analysis
Source: Front Psychiatry. 2023 May 17;14:1144413. doi: 10.3389/fpsyt.2023.1144413 (PMC10229880; doi:10.3389/fpsyt.2023.1144413)

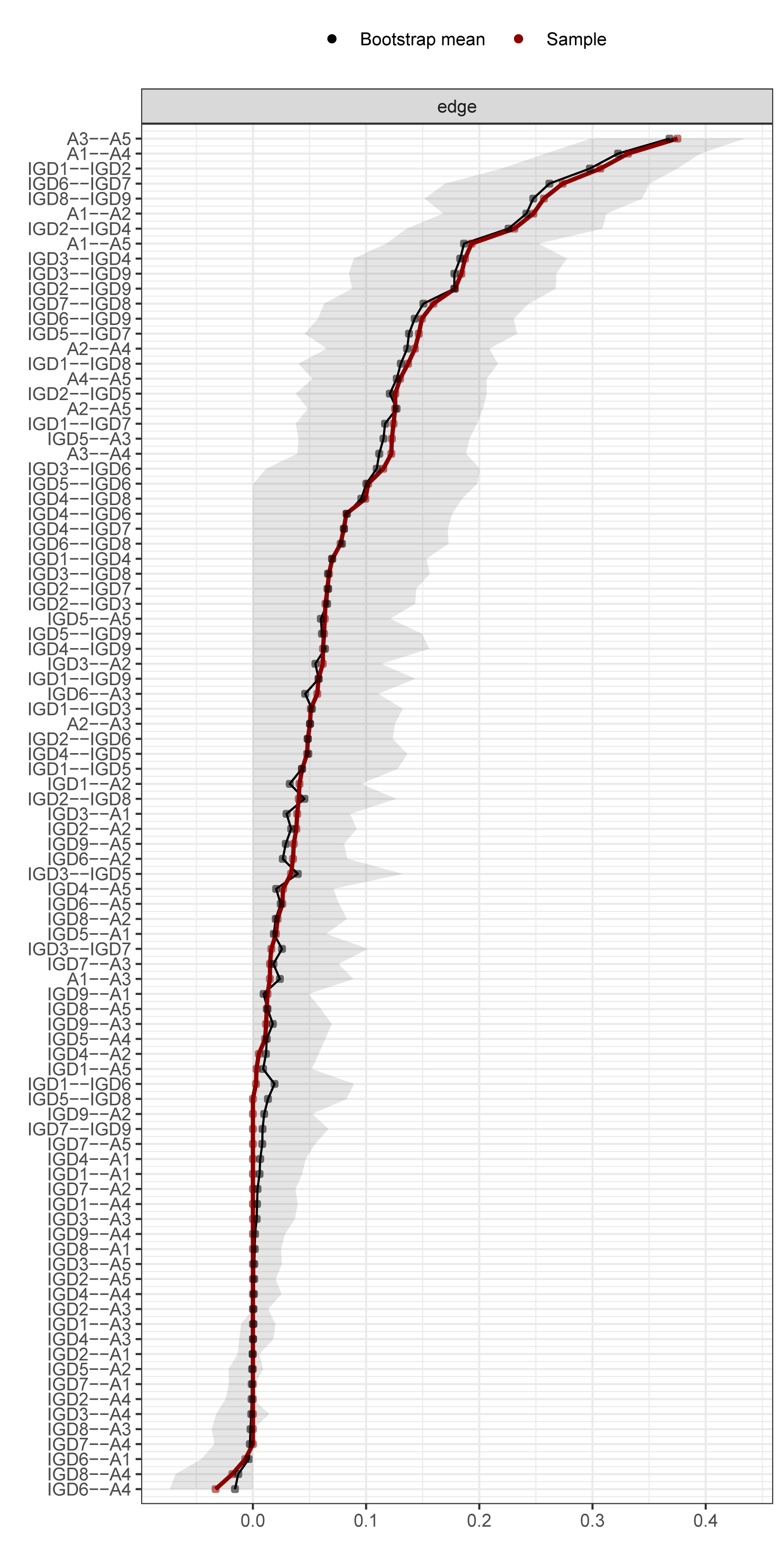

Supplement: Supplementary Figure 1 — Accuracy test of edge weights in the anxiety-IGD network. The red line indicates the sample edge weight values and the gray area indicates the bootstrapped confidence intervals. A1, separation anxiety; A2, social phobia; A3, panic disorder; A4, physical injury fear; A5, generalized anxiety; IGD1, preoccupation; IGD2, tolerance; IGD3, giving up other activities; IGD4, continuing despite problems; IGD5, escape; IGD6, negative consequences; IGD7, loss of control; IGD8, deception; IGD9, withdrawal. [file Image_1.tif]

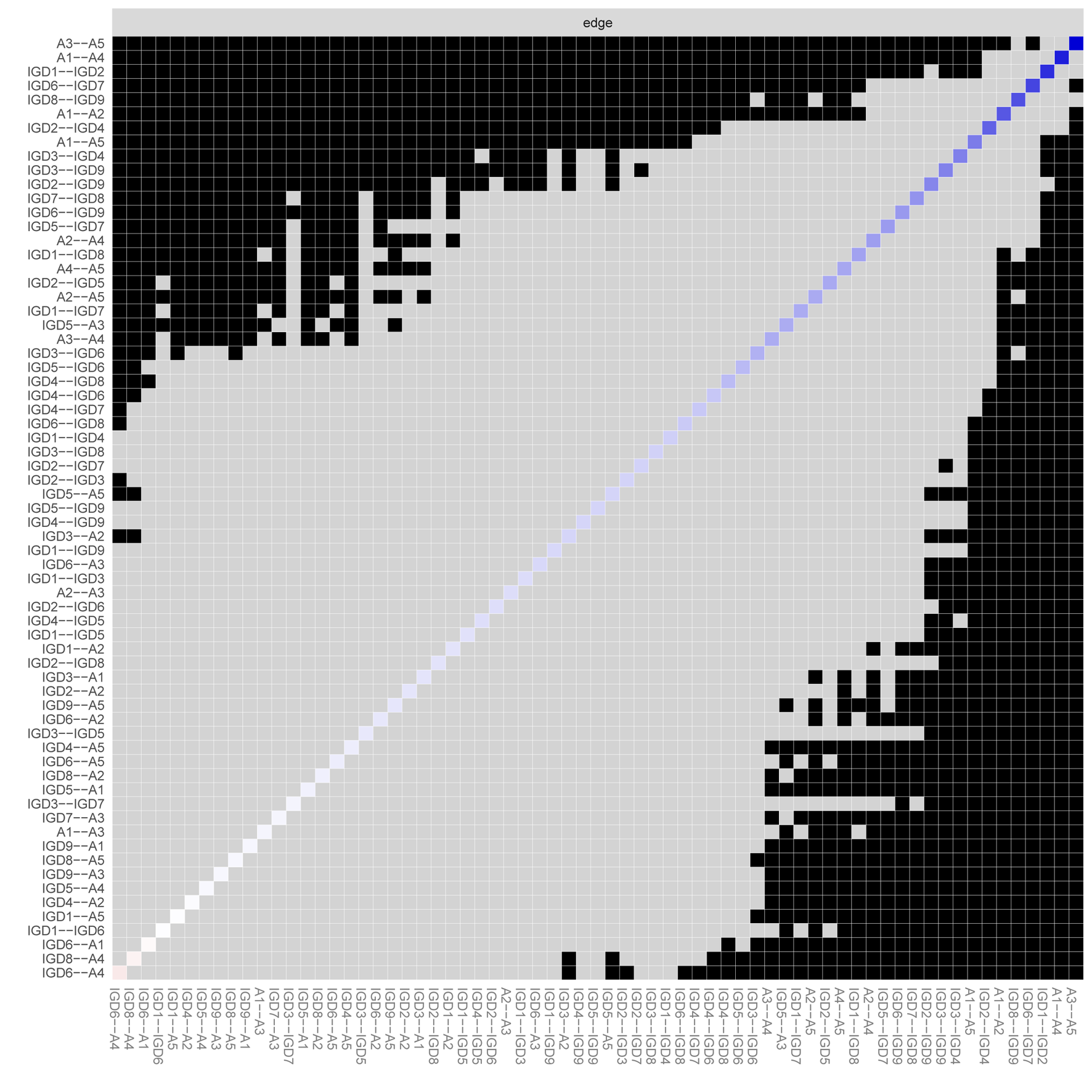

Supplement: Supplementary Figure 2 — Bootstrapped difference test of edge weights in the anxiety-IGD network. The black box indicates that the edge weights of the two corresponding node pairs have a significant difference, the gray box indicates no significant difference. Blue and red boxes on the diagonal correspond to edge weights with positive and negative correlations, respectively. A1, separation anxiety; A2, social phobia; A3, panic disorder; A4, physical injury fear; A5, generalized anxiety; IGD1, preoccupation; IGD2, tolerance; IGD3, giving up other activities; IGD4, continuing despite problems; IGD5, escape; IGD6, negative consequences; IGD7, loss of control; IGD8, deception; IGD9, withdrawal. [file Image_2.tif]

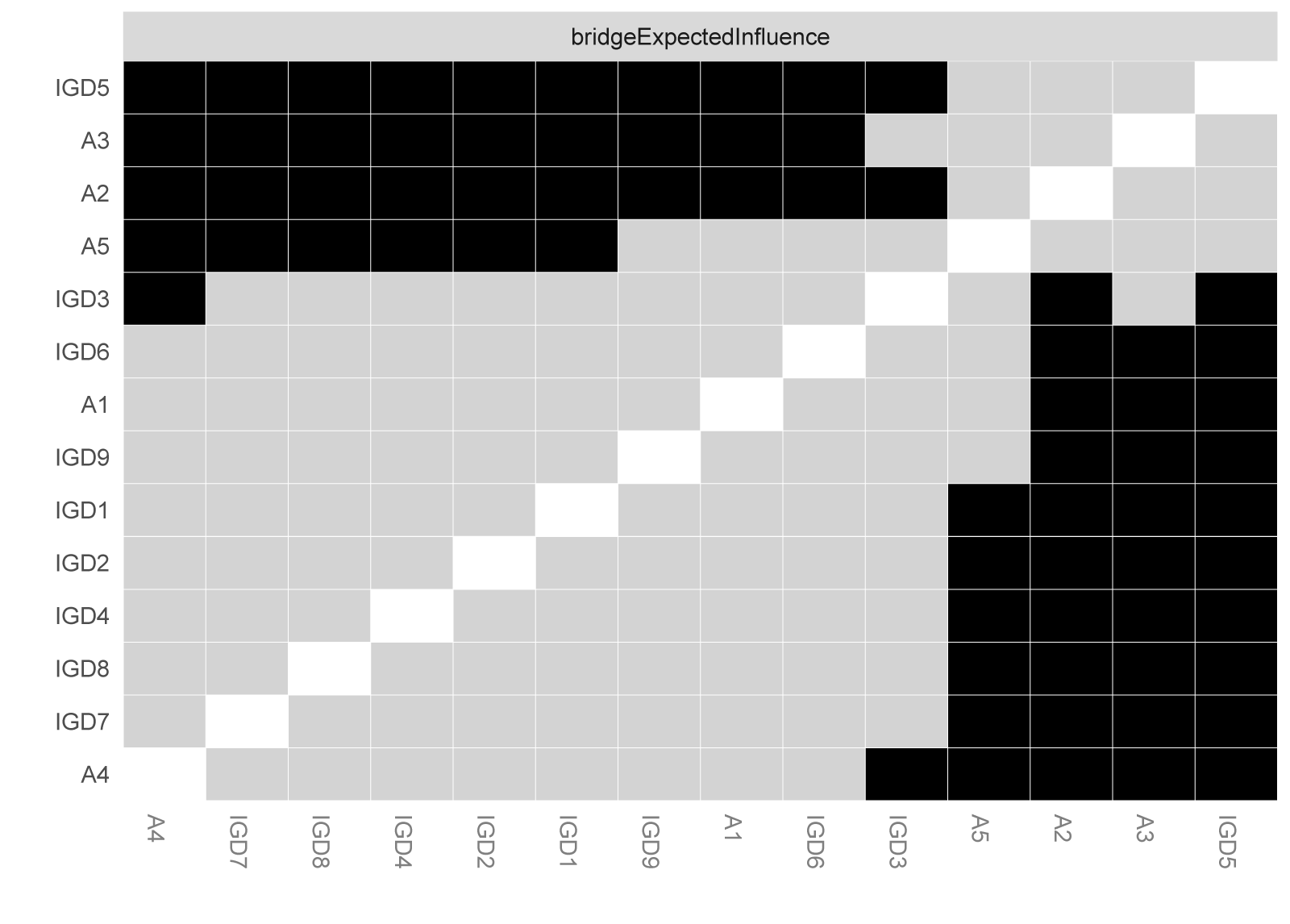

Supplement: Supplementary Figure 3 — Bootstrapped difference test of bridge expected influences in the anxiety-IGD network. The black box indicates that the bridge expected influences of the two corresponding nodes have a significant difference, the gray box indicated no significant difference. A1, separation anxiety; A2, social phobia; A3, panic disorder; A4, physical injury fear, A5, generalized anxiety, IGD1, preoccupation; IGD2, tolerance; IGD3, giving up other activities; IGD4, continuing despite problems; IGD5, escape; IGD6, negative consequences; IGD7, loss of control; IGD8, deception; IGD9, withdrawal. [file Image_3.tif]

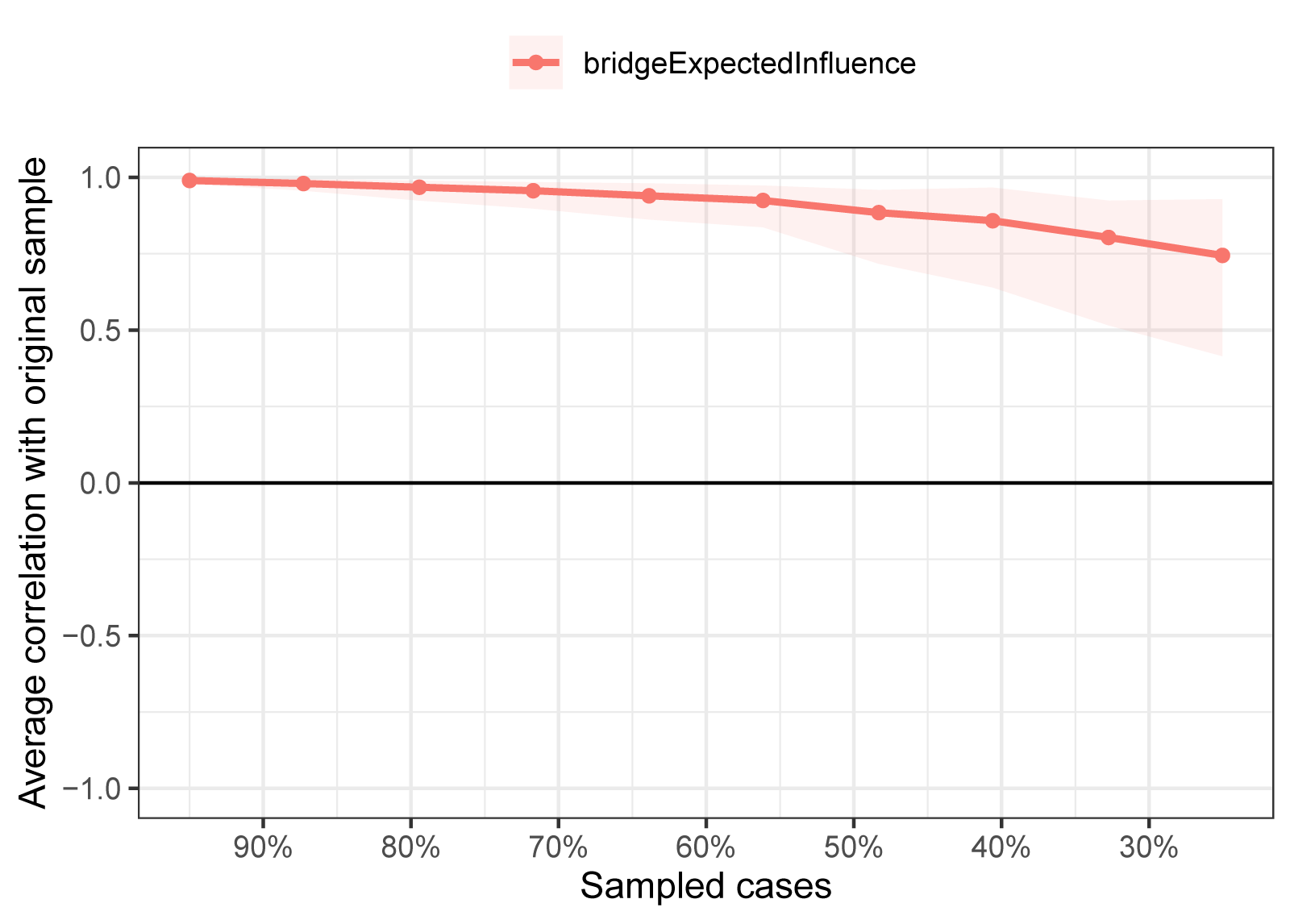

Supplement: Supplementary Figure 4 — Stability of bridge expected influences in the anxiety-IGD network. The red bar represents the average correlation between bridge expected influences in the full sample and subsample with the red area depicting the 2.5th quantile to the 97.5th quantile. [file Image_4.tif]
